# The effect of application of digestate and agro-food industry sludges on Dystric Cambisol porosity

**DOI:** 10.1371/journal.pone.0238469

**Published:** 2020-09-02

**Authors:** Kamil Skic, Zofia Sokołowska, Patrycja Boguta, Anna Skic

**Affiliations:** 1 Department of Physical Chemistry of Porous Materials, Institute of Agrophysics, Polish Academy of Sciences, Lublin, Poland; 2 Department of Mechanical Engineering and Automatic Control, University of Life Sciences, Lublin, Poland; Lappeenranta-Lahti University of Technology (LUT University), FINLAND

## Abstract

The spatial arrangement and pore size distribution play an important role in accumulation and protection of exogenous organic matter (EOM) in the soil, but how different organic materials contribute to modify pore structure is poorly understood. We aimed at exploring possible changes in the complexity of the soil phase during fertilization with different doses of digestate and sludges sourced from the agro-food industry. For this purpose, the short-term effects—one year, of soil fertilization, were investigated in several sampling periods and within two depths (0–25 cm and 25–40 cm). Changes in the specific surface area (SSA), total pore volume (V_MIP_), total pore area (S_MIP_), average pore radius (R_MIP_) and pore size distribution (PSD) were monitored using N_2_ adsorption/desorption (NAD) and mercury porosimetry (MIP) methods. Our results showed that the intensity of observed changes depended on the type and dose of organic material, soil depth and sampling date. Accumulation of EOM increased with soil depth, masking a large proportion of SSA. Deeper soil layer was more susceptible to changes in the pore size distributions due to the formation of new elongated pores. We concluded that this specific structural porosity was related to the decomposition of organic matter during the formation of soil aggregates.

## Introduction

Increasingly intense agricultural production and food industry businesses leads to a gradual increase in the amount of different organic wastes. Among them, agro-industrial wastes can be distinguished. In line with Pascual et al. [1], they are solid or liquid materials generated from the direct consumption of primary products or industrialization. Over the years 2010–2018, production of industrial sludge in Poland increased by 20% and reached 463 million tons in 2018. These constitute 44% of the total amount of sludge produced in Poland, estimated at 1046.5 million tons of dry solids in a given year. According to the Central Statistical Office, most of the industrial sludge were landfilled (23.4%) or thermally transformed (26.5%), and only 5.8% was used in agriculture and for reclamation purposes.

Sustainability requires organic waste to be managed as a valuable resource, rather than as a strain for the environment [2]. The most promising and appropriate way to properly manage and reuse wastes of industrial or agricultural origin is soil fertilization. Table 1 presents the influence of various organic waste on soil physical, chemical and biological properties as well as on crop parameters.

**Table 1:** Crop parameters and physical, chemical and biological responses of soil amended with organic wastes.

Properties	Effect	References
**Physical**		
Aggregate stability	Increase	[3–8]
	Decrease	[9]
Water holding capacity	Increase	[6, 7, 9]
	No change	[10]
Porosity	Increase	[8, 11]
Bulk density	Decrease	[4, 6, 11]
Specific surface area	Decrease	[12]
**Chemical**		
pH	Increase	[13]
	Decrease	[2, 10, 11, 14]
	No change	[5]
Heavy metals (Cd, Cr, Ni, Pb etc.)	Increase	[10, 14–17]
	Decrease	[18]
Macronutrients (N, P, K)	Increase	[10, 11, 16, 19]
Micronutrients (Fe, Zn, Cu, Mn etc.)	Increase	[16]
	Decrease	[13]
Exchangeable sodium percentage	Increase	[13]
	Decrease	[4]
Organic matter	Increase	[6, 7, 11, 15, 17]
Organic carbon	Increase	[3, 4, 9, 12, 14, 16, 18]
	No change	[20, 21]
Humic acid	Increase	[19]
Electrical conductivity	Increase	[2, 5, 10, 13, 14, 21]
	Decrease	[11]
Cation exchange capacity	Increase	[11]
	Decrease	[13]
**Biological**		
Enzyme activities	Increase	[7, 18, 21]
Microbial biomass	Increase	[5, 7, 18]
Microbial activity	Increase	[17, 21, 22]
	Decrease	[20]
**Crop parameters**		
Plant biomass	Increase	[2, 14, 15, 23]
Crop yield	Increase	[2, 6, 13, 16, 23, 23, 24]
Heavy metals (Cd, Cr, Ni, Pb etc.)	Increase	[11, 14, 16]

The potential benefits that come from the reuse of organic waste in agricultural land include supplying nutrients (nitrogen, phosphorus, potassium and micronutrients) to the crops [10], improving soil fertility, and increasing soil organic matter content [17]. Moreover, organic waste enhances soil physical properties such as bulk density [4], water-holding capacity [9], and stability of soil aggregates [3]. It is especially important in the case of soils exposed to erosion, compaction, as well as management practices that lead to the gradual depletion of carbon compounds [18]. On the other hand, some authors reported negative aspects of the agricultural application of organic wastes. In line with the literature, they include: raising the levels of heavy metals in the soil and vegetation [14] increase in soil salinity [13], reduction in microbial activity [25], greenhouse gas emissions and the release of odorous compounds [26, 27]. Due to the above adverse effects and possible food chain contamination, a regular monitoring of soil and plant parts properties should be taken up while organic waste continuous use.

Among many publications concerning the effect of the organic waste application on agricultural land, only a small percentage deals with soil physical properties, including soil porosity. Soil porosity affects soil processes such as the movement of air, water and other fluids [28, 29], water retention [6], availability of nutrients and microbial activity [30]. Spatial arrangement and pore size distributions play also an important role in the long-term organic carbon storage and its availability in soil [31]. Organic fertilization may enhance soil organic matter stocks by filling the pores in micro- and macroaggregates or in larger cracks between soil particles [8, 31]. Some laboratory and field experiments demonstrated that the application of organic matter increased the number of larger pores in soils and promoted the formation and stabilization of biogenic macropores [32, 33]. Other authors showed a reduction in micropores as a result of the application of industrial sludge [12]. Given the preferential changes discussed above the knowledge on the effect of organic matter from exogenous sources on soil pore structure is required.

There is not enough quantitative description of changes in the soil pore system after fertilization with different types of organic waste, especially by industrial sludge. Understanding the process of organic matter accumulation in soil and its relation to application rates needs both short- and long-term observations. This work aimed to assess the influence of different organic wastes application on pore characteristics of cultivated soil. The short-term (one year) effects of soil fertilization with digestate from an agricultural biogas plant and sludges from the agro-food industry were analysed within two soil depths. The soil pore characteristics were gathered using the nitrogen adsorption/ desorption (NAD) and mercury intrusion porosimetry (MIP) methods. The adsorption of nitrogen primarily allowed the determination of specific surface area (SSA). In turn, MIP was used to determine pore size distribution (PSD), total pore volume (V_MIP_), average pore radius (R_MIP_), and total pore area (S_MIP_).

## Materials and methods

### Study site and experimental design

The field experiment was set up at a site located in the vicinity of the town of Krasnystaw, south-east Poland. The soil was classified as a Dystric Cambisols, according to the WRB classification. The field studies did not involve endangered or protected species. The land was privately owned and, before the experiment started, its management had been conventional with annual ploughing. The owner of the land gave permission to conduct the study on this site. In the year of the experiment, the average monthly temperatures did not differ significantly from the average monthly temperatures estimated over the decade. The average annual temperature did not exceed 8.2°C, with amplitudes of 28.2°C. Annual rainfall was 512 mm. The experimental area was divided into plots with an area of 10 m^2^ surrounded by a 1 m wide protection buffer. Exogenous organic matter (EOM) of different origin and physicochemical properties, namely digestate (PS), dairy sludge (DS), and fruit sludge (FS), were used as fertilizers and introduced into the soil in two doses: 4.5 and 9.0 Mg of dm. per hectare. The maximum dose was set in accordance with the requirements of the Polish regulations on the use of waste on land and waste management. The organic material was spread evenly over the soil surface and ploughed to a depth of 25 cm. The control object was the soil without any addition of organic material. The plots were sown with wheat, spring variety Kandela. During the year-long experiment, samples were taken four times (t_1_: April–control term, t_2_-t_4_: May, July, August, respectively) from the depths of 0–25 cm and 25–40 cm. Three plots were used as replicates in each variant. The sampling locations on each plot were chosen randomly. The soil samples were air-dried and sieved through a 2 mm mesh. Visible fractions of roots and mesofauna were removed before further investigation.

### Waste characteristics

PS was produced at an agricultural biogas plant. The basic substrates in the dry fermentation process (at 48°C) were silage from corn and rye, apple pomace, and distillery decoction. After an anaerobic process, PS was kept in tanks for three months, and then was applied in the experimental field in liquid form. In contrast, FS and DS were the by-products of mechanical and aerobic treatment of wastewater generated during the preparation of dairy and fruit products and during the washing of individual productions lines. Since the production was specialized, the wastewater was not changing over time. Their main biochemical characteristics provided by the producer were as follows: fruit wastewater treatment plant—BOD_5_ (biological oxygen demand): 200–1450 mg O_2_∙dm^-3^, COD (chemical oxygen demand): 300–2000 mg O_2_∙dm^-3^, general suspensions: 500–2500 mg∙dm^-3^; dairy wastewater treatment plant: BOD_5_: 2000–3500 mg O_2_∙dm^-3^, COD: 300–5000 mg O_2_∙dm^-3^, general suspensions: 300–1200 mg∙dm^-3^. The amount of sludge generated during wastewater purification each year is estimated at 150 Mg per year for FS and at about 2000 Mg per year for DS.

### Physicochemical analyses of soil and wastes

The characteristics of soil samples and waste materials were gathered as follows: the granulometric composition of soil samples was estimated in accordance with the Casagrande-Prószyński method; pH was measured in 1:5 (v/v) suspensions using a digital pH-meter (CX-505, Elmetron); the total carbon content (TC) and organic carbon content were determined for the dried samples of about 1g by their combustion in 1250 ºC (TOC MULTI N/C 2000, HT 1300, Analytic Jena); the ash content was determined by sample combustion in 550 ºC for six hours in a muffle furnace (FCF 12 SP, Czylok); solid phase density was determined using a helium pycnometer (Ultrapycnometer 1000, Quantachrome Corp); total nitrogen in soil samples was estimated in accordance with the Kjeldahl method and phosphorus easily available for plants by the Egner-Riehm method; the measurement of the total content of Cd, Zn, Pb, Cu, Ni, Cr, Ca, Mg, K was performed using atomic absorption spectrometry (AAS contra AA 300, Analytic Jena); the concentration of Fe_2_O_3_, Al_2_O_3_ in soil samples was measured using a desktop XRF crystal diffraction scanning spectrometer (SPECTROSCAN MAKC-GV1) [34].

### Mercury intrusion analysis

An Autopore IV 9500 (Micrometrics, USA) mercury porosimeter was used to determine the pore size distribution in the range from 0.003 μm to 360 μm in diameter. All measurements were performed on dried samples (105°C, 24 hours) of mass of approximately 0.5 g. The volume of mercury introduced into the sample at a specific pressure corresponds to the volume of available pore throats. The intrusion pressure was converted into an equivalent pore radius (R) following the Washburn equation

$$P=‐(A\sigma_{Hg}cos\alpha_{Hg})/R$$ (1)

where P is the external pressure (Pa), σ_Hg_ is the mercury surface tension (48.9 J∙m^−2^), α_Hg_ is the mercury/solid contact angle (taken as 141.3°) and A is a shape factor (equal to 2 for the assumed shape of pores). The average pore radius (R_MIP_) was obtained assuming that all pores are right cylinders, thus, when the volume (V_MIP_ = πr2L) is divided by the pore area (S_MIP_ = 2πrL), the R_MIP_ will be equal to 2V_MIP_ / S_MIP_.

### Nitrogen adsorption at 77K

Nitrogen adsorption/desorption isotherms were obtained with a surface characterization analyzer, 3Flex (Micromeritics, USA). Before measurements, the soil samplesere heated at 105°C overnight under vacuum to remove water and gaseous contaminants. The calculation of SSA was based upon the adsorptive branch of the isotherm as applied to the Brunauer, Emmett and Teller equation for multilayer adsorption:

$$\left(p/p_{0})/(N(1‐p/p_{0})=1/(N_{m}C)+\left(C‐1\right)/(N_{m}C)(p/p_{0}\right)$$ (2)

where p and p_0_ are the equilibrium and the saturation pressure of the adsorbate, respectively, N is the volume adsorbed at the pressure p/p_0_, N_m_ is the volume of adsorbate corresponding to specific monolayer capacity, and C is a constant related exponentially to the enthalpy (heat) of adsorption in the first adsorbed layer.

In the first step of calculation, the linear form of BET equation in the range of approximately 0.05 to 0.30 p/p_0_ was used to obtain a monolayer capacity. Then, having learnt the nitrogen quantity required to form a monolayer on the surface, SSA cabe calculated from N_m_ assuming that the monolayer has a close-packed structure giving the nitrogen molecule area of 0.162 nm^2^ at 77K. Considering the foregoing, SSA is estimated from the following dependence

$$SSA=N_{m}L\sigma_{m}/M$$ (3)

where SSA is the BET specific surface area of the adsorbent (of mass m), σ_m_ is the cross-sectional adsorbate area, M is the molecular weight of the adsorbate, and L is Avogadro's number.

### Statistical analysis

All measurements were in triplicate, and the statistical analysis was performed using Statistica 13.1 (Dell Inc.). The one-way analysis of variance (ANOVA) with post-hoc analysis (HSD Tukey test) were performed to test the differences for measured parameters: TC, SSA, R_MIP_, V_MIP_ and S_MIP_. The tests were carried out separately for each exogenous material, applied dose, as well as soil depth. The differentiating factor was the period of sampling. The significance level was estimated at α = 0.05, n = 12. The assumption of normal distribution for all data used in statistical calculations was verified by the Shapiro-Wilk test.

To check the influence of TC on the parameters of SSA, V_MIP_, R_MIP_ and S_MIP_ for particular soil depths, Pearson’s correlation coefficients analysis was used. The mean values of PS, DS, and FS variants (4.5 and 9.0 Mg·ha^-1^) from each term were used together in the analysis (n = 24). The significance level was estimated at α = 0.05.

For abbreviations used in the materials and method section and subsequent sections refer to Table 2.

**Table 2:** The list of abbreviations.

DS	Dairy sludge
DM	Dry mass
EOM	Exogenous organic matter
FS	Fruit sludge
MIP	Mercury porosimetry
NAD	Nitrogen adsorption/ desorption
PS	Digestate
PSD	Pore size distributions
R_MIP_	Average pore radius
S_MIP_	Total pore area
SSA	Specific surface area
TC	Total carbon content
TN	Total nitrogen content
TOC	Total organic carbon
V_MIP_	Total pore volume

## Results

### Physicochemical properties of studied soil and wastes

Obtained results showed that the soil was slightly acidic and poor in macro-elements such as N, K, P, Ca and Mg. The content of heavy metals did not exceed the level that could limit the use of waste materials for fertilizing purposes. The soil was characterized by a low content of organic and total carbon, which decreased with depth. Soil at the depth of 25–40 cm contained a slightly higher amount of clay, aluminium and iron oxides, compared to the depth of 0–25 cm. The basics soil properties obtained for the two soil depths are presented in Table 3.

**Table 3:** Soil characteristics obtained for the depths of 0–25 cm and 25–40 cm at the beginning of field experiment.

	Depth 0–25 cm		Depth 25–40 cm	
**Sand (%)**	9	± 1.02	4	± 0.63
**Silt (%)**	54	± 0.98	52	± 1.15
**Clay (%)**	37	± 0.56	44	± 1.29
**Ash (%)**	97.9	± 0.9	98.2	± 0.6
**Particle density (g·cm**^**-3**^**)**	2.62	± 0.15	2.65	± 0.01
**pH (H**_**2**_**O)**	6.80	± 0.10	6.34	± 0.11
**pH (KCl)**	5.38	± 0.14	5.09	± 0.11
**TOC (g·kg**^**-1**^ **d.m.)**	9.22	± 0.18	1.57	± 0.11
**TC (g·kg**^**-1**^ **d.m.)**	11.5	± 0.93	3.36	± 0.21
**TN (g·kg**^**-1**^ **d.m.)**	0.83	± 0.02	0.27	± 0.01
**P**_**2**_**O**_**5**_ **(mg P**_**2**_**O**_**5**_**/100g)**	9.70	± 0.49	2.20	± 0.07
**K (g·kg**^**-1**^ **d.m.)**	0.17	± 0.01	0.19	± 0.01
**Mg (g·kg**^**-1**^**d.m.)**	0.30	± 0.01	0.38	± 0.02
**Ca (g·kg**^**-1**^**d.m.)**	0.43	± 0.02	0.44	± 0.03
**Zn (mg·kg**^**-1**^**d.m.)**	20.0	± 0.60	20.0	± 0.8
**Cu (mg·kg**^**-1**^**d.m.)**	0.86	± 0.02	1.19	± 0.06
**Cd (mg·kg**^**-1**^**d.m.)**	0.03	< 0.01	< 0.01	-
**Ni (mg·kg**^**-1**^**d.m.)**	1.67	± 0.07	1.68	± 0.08
**Pb (mg·kg**^**-1**^**d.m.)**	10.5	± 0.31	10.3	± 0.2
**Cr (mg·kg**^**-1**^**d.m.)**	<0.078	-	<0.078	-
**Al**_**2**_**O**_**3**_ **(g·kg**^**-1**^**d.m.)**	77.8	± 0.77	99.2	± 3.02
**Fe**_**2**_**O**_**3**_ **(g·kg**^**-1**^**d.m.)**	21.0	± 0.52	33.5	± 2.23

The table presents mean values ± standard deviation, n = 3. Abbreviations: TOC—organic carbon, TC—total carbon, TN—total nitrogen.

The basics physicochemical properties of waste materials are presented in Table 4. Evidently, they contained substantial amounts of carbon and nitrogen compounds that constituted their fertilizer value. PS was neutral (pH around 7.3) as opposed to DS and FS that were medium acidic (pH around 6.0). The studied materials were also characterized by low dry matter content, medium or low content of macro and microelements, as well as the narrow C/N ratio. Waste materials met the requirements for environmental management in terms of heavy metal concentration and the presence of bacteria and parasite eggs.

**Table 4:** Characteristics of sludge from a dairy wastewater treatment plant (DS), a fruit industry wastewater treatment plant (FS) and digestate from an agricultural biogas plant (PS).

	PS		FS		DS	
**DM (%)**	5.20	± 0.26	18.7	± 0.9	13.0	± 0.3
**Ash (%)**	20.9	± 1.3	30.0	± 0.6	12.9	± 0.7
**Particle density (g·cm**^**-3**^**)**	1.57	± 0.06	1.62	± 0.08	1.32	± 0.04
**pH (H**_**2**_**O)**	7.29	± 0.26	5.99	± 0.24	6.06	± 0.10
**pH (KCl)**	6.82	± 0.14	5.98	± 0.15	5.90	± 0.20
**TOC (g·kg**^**-1**^ **d.m.)**	372	± 7	343	± 5	479	± 11
**TC (g·kg**^**-1**^ **d.m.)**	496	± 10	392	± 12	629	± 25
**TN (g·kg**^**-1**^ **d.m.)**	49.3	± 0.25	43.5	± 0.83	84.1	± 0.76
**C:N**	7.54		7.88		5.69	
**P (g·kg**^**-1**^ **d.m.)**	14.4	± 0.3	5.90	± 0.24	15.2	± 0.8
**K (g·kg**^**-1**^ **d.m.)**	11.6	± 0.47	3.92	± 0.12	20.5	± 0.4
**Mg (g·kg**^**-1**^ **d.m.)**	7.17	± 0.29	4.05	± 0.16	5.98	± 0.30
**Ca (g·kg**^**-1**^ **d.m.)**	19.3	± 0.6	7.62	± 0.31	11.2	± 0.7
**Zn (mg·kg**^**-1**^ **d.m.)**	1450	± 58	1500	± 45	280	± 11
**Cu (mg·kg**^**-1**^ **d.m.)**	134	± 5	80.3	± 1.6	11.3	± 0.6
**Cd (mg·kg**^**-1**^ **d.m.)**	2.47	± 0.07	1.70	± 0.07	0.49	± 0.03
**Ni (mg·kg**^**-1**^ **d.m.)**	28.8	± 1.2	29.2	± 1.2	3.49	± 0.18
**Pb (mg·kg**^**-1**^ **d.m.)**	39.7	± 1.6	27.8	± 0.8	10.3	± 0.2
**Cr (mg·kg**^**-1**^ **d.m.)**	<0.078	-	<0.078	-	<0.078	-

The table contains mean values ± standard deviation, n = 3. Abbreviations: DM—dry mass, TOC—organic carbon, TC—total carbon, TN—total nitrogen.

### Nitrogen adsorption-desorption isotherms

**Fig 1:**
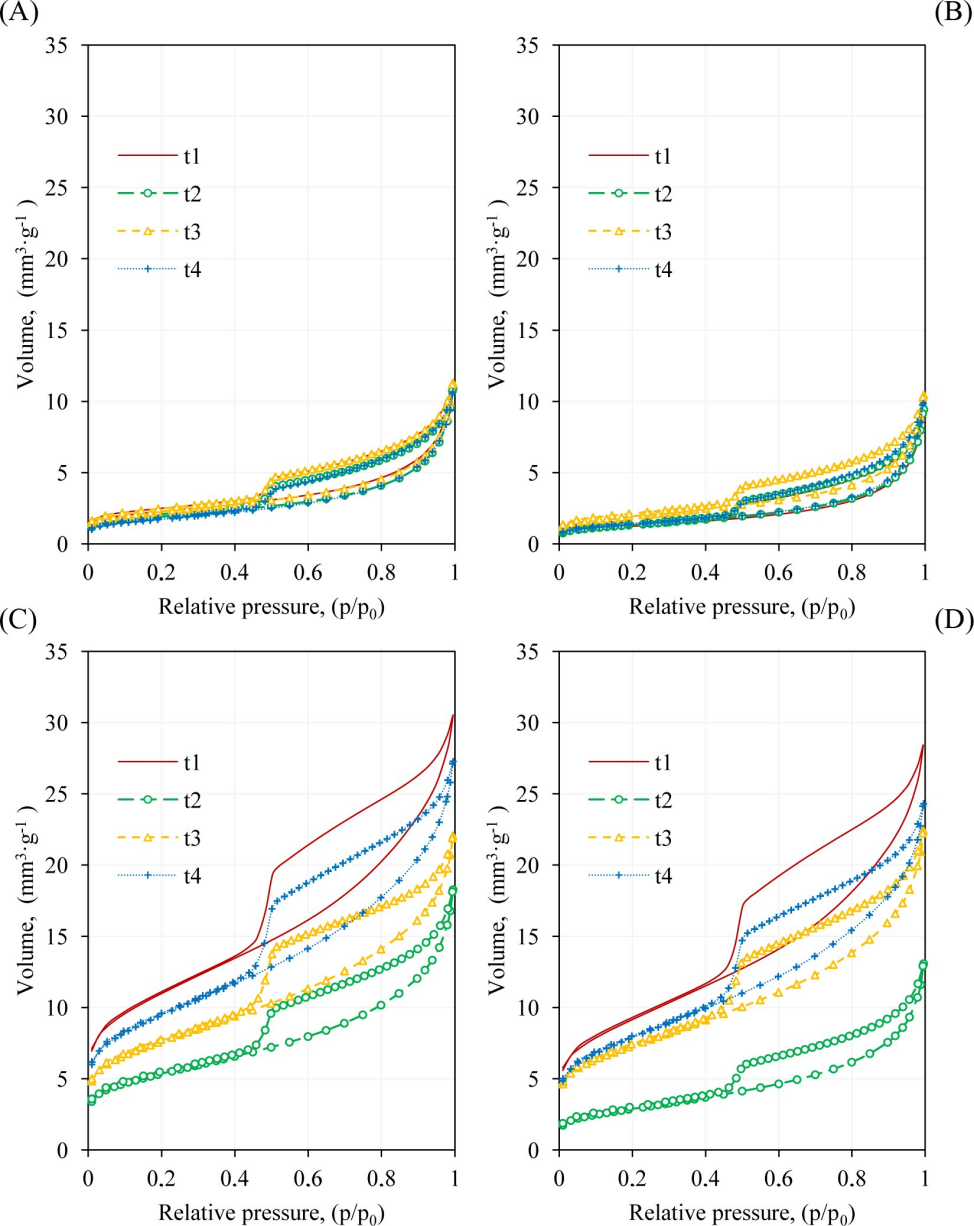
N_2_ isotherms obtained from soil treated with sludge from a dairy wastewater treatment plant (DS). (A) DS, soil depth 0–25 cm, dose 4.5 Mg/ha; (B) DS, soil depth 0–25 cm, dose 9.0 Mg/ha; (C) DS, soil depth 25–40 cm, dose 4.5 Mg/ha; (D) DS, soil depth 25–40 cm, dose 9.0 Mg/ha. Abbreviations: t_1_- control, t_2_-t_4_- sampling terms after fertilization.

Examples of nitrogen isotherms obtained at two soil depths modified with two doses of sludge are presented in Fig 1. All curves belonged to type II and had the shape typical of the physical adsorption process [35]. The isotherms were characterized by the presence of a small inflexion in the range of low relative pressure (p/p_0_ ~ 0.05), more or less inclined middle section (p/p_0_ from ~ 0.05 to ~ 0.9), and a rapid increase in the amount adsorbed at p/p_0_ ~ 0.9 without the saturation effect. The amount of gas adsorbed at low p/p_0_ values corresponded to the volume of micropores and fine mesopores, whereas at higher relative pressure, it was related to the volume of larger mesopores and macropores. The adsorption/desorption isotherms overlapped at low relative pressures while appeared as hysteresis at p/p_0_> 0.42. The shape of hysteresis loops with unrestricted adsorption at high relative pressures allowed their classification as type H3. This type of hysteresis loop is characteristic especially of aggregates with a predominance of plate-like particles that produce slit-shaped pores [36].

The application of exogenous organic matter to soil caused changes in the shape and course of N_2_ isotherms only at the soil depth of 25–40 cm. All fertilized variants caused lower adsorption values and a shift of isotherms, accompanied by the narrowing of the hysteresis loops. The amount of N_2_ adsorption differed greatly depending on the period of sampling and the dose and type of exogenous organic material. The largest decrease in the volume of adsorbed gas was observed in the second sampling period and for a higher dose of PS and DS.

### Specific surface area

**Fig 2:**
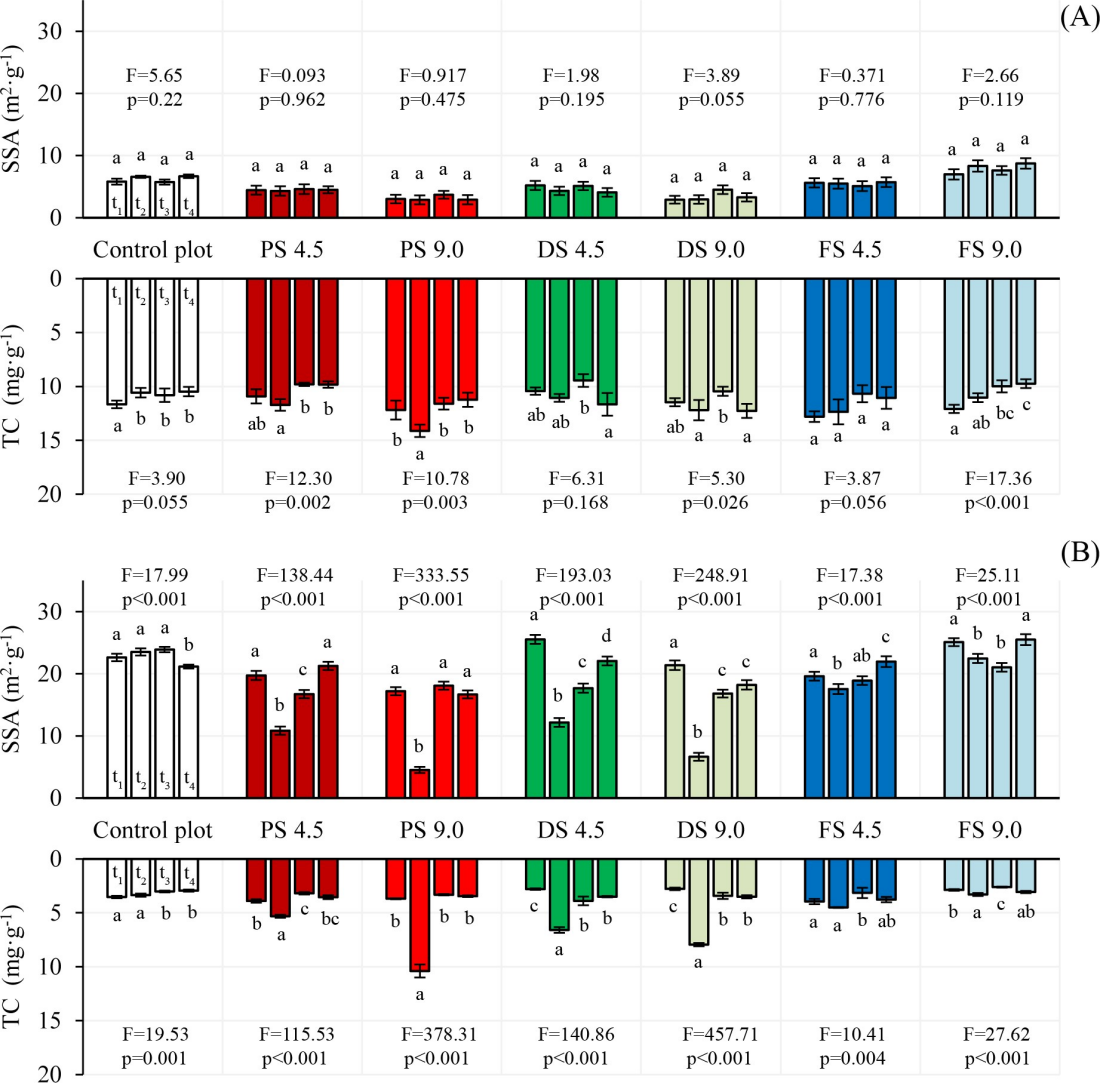
The dynamic of changes in SSA and TC during particular experiment periods. (A) soil depth 0–25 cm, (B) soil depth 25–40 cm. The figure shows mean values ± standard deviation. The same letter means no significant differences between the values at the level of significance α = 0.05, one-way ANOVA variance analysis, Tukey's HSD test. Abbreviation: PS—digestate from an agricultural biogas plant, DS—sludge from a dairy wastewater treatment plant, FS—sludge from a fruit wastewater treatment plant, t_1_-t_4_—means the period of sampling.

Quantitative differences in nitrogen adsorption were expressed in the calculation of SSA and compared with TC. We decided to choose the TC parameter rather than organic carbon due to the substantial amount of carbonates in waste material. The changes in the investigated parameters during particular experiment periods are presented in Fig 2. The average values of SSA and TC, obtained for control and fertilized variants at a soil depth of 0–25 cm, ranged from 2.88 to 8.74 m^2^/g and from 9.45 mg/g to 14.12 mg/g respectively. In turn, for the depth of 25–40 cm, they fell within the range from 4.52 to 25.53 m^2^/g for SSA and from 2.61 mg/g to 10.39 mg/g for TC.

In the case of the depth of 0–25 cm, we did not observe any significant changes in the parameter of SSA (Fig 2A). The TC content increased in the second period of the experiment (t_2_) for the soil with the addition of a higher dose of PS and DS, 16% and 6%, respectively. In the case of PS, the differences were statistically significant. The FS initially caused a decreasing trend in the TC parameter. The observed changes were close to those for the control, i.e. without EOM application.

The application of PS, DS and FS to soil caused a significant decrease of SSA and an increase of TC for a soil depth of 25–40 cm and for almost all tested variants (Fig 2B). The effect of EOM application was the most evident in the second period of experiment (t_2_) and for objects with a higher dose (9 Mg/ha) of PS and DS. In these cases, the SSA values decreased from 17.2 m^2^/g to 4.52 m^2^/g (74%) and from 21.38 m^2^/g to 6.65 m^2^/g (69%), while TC increased from 3.69 mg/g to 10.39 mg/g (181%) and from 2.78 mg/g to 7.95 mg/g (186%), respectively, for PS and DS variants. The application of a higher dose of FS affected the SSA parameter to a minor extent. SSA decreased just from 25.0 to 22.5 m^2^/g (10%) with TC increasing from 2.88 to 3.30 (15%). The lower dose of EOM (4.5 Mg/ha) also influenced SSA and TC. The observed changes were smaller compared to the higher dose. The average values of SSA decreased as follows: 8.89 m^2^/g (45%) for PS, 13.36 m^2^/g (52%) for DS and 2.07 m^2^/g (11%) for FS.

In further experiment periods (t_3_ and t_4_), at the depth of 25–40 cm, SSA generally started to increase as TC decreased. This effect was clearly visible only in variants with PS and DS.

**Fig 3:**
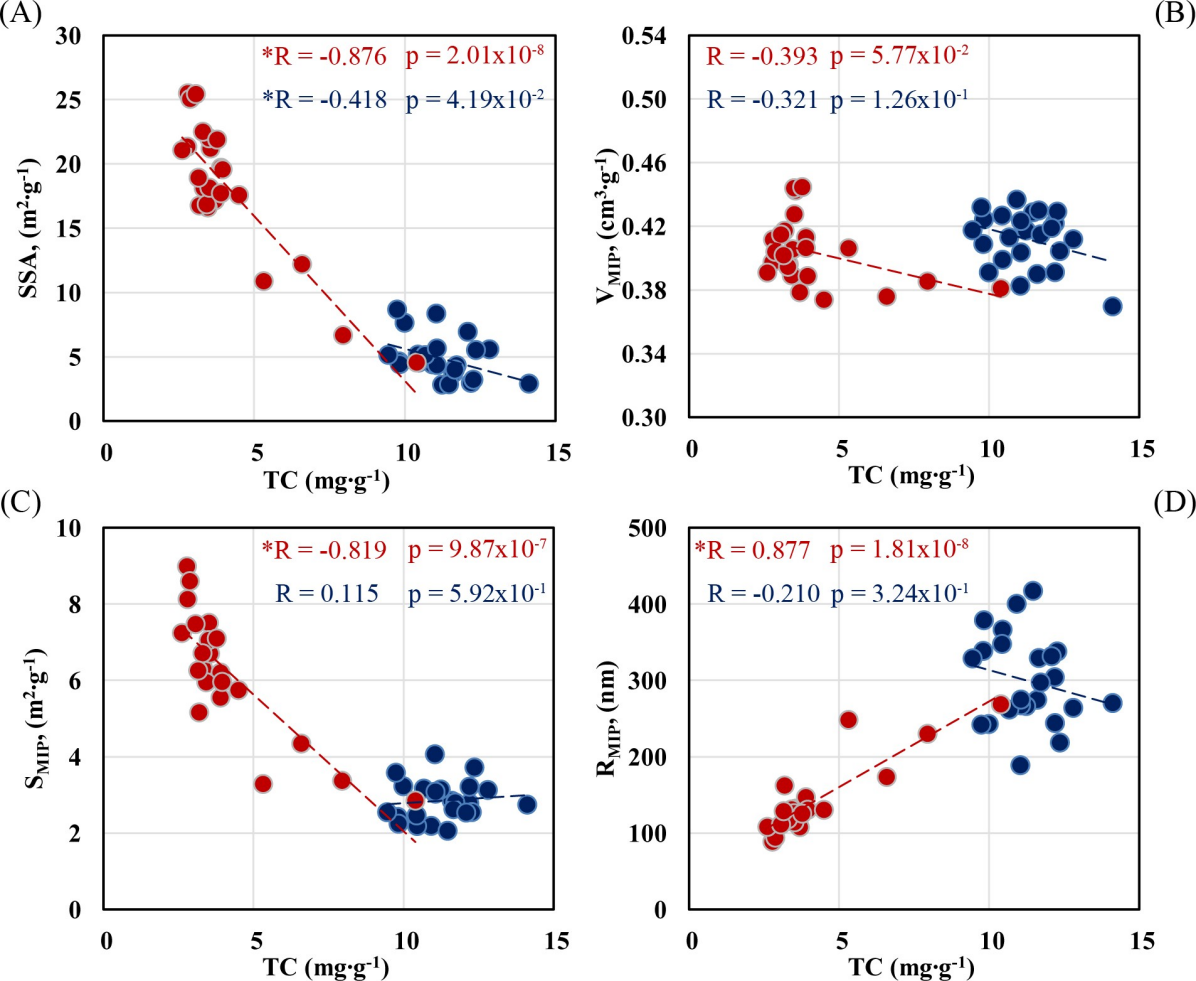
Pearson’s correlations between the tested parameters. (A) TC versus SSA, (B) TC versus V_MIP_, (C) TC versus S_MIP_, (D) TC versus R_MIP_. The blue dots refer to a soil depth of 0–25 cm, the red dots refer to a soil depth of 25–40 cm. The asterisk indicates the significance of the results. The significance level was evaluated at α = 0.05, n = 24. Abbreviations: TC–total carbon content, SSA–specific surface area, V_MIP_−total volume of pores, S_MIP_−total pore area, R_MIP_−average pore radius.

Pearson’s correlations between the tested parameters are shown in Fig 3. The correlations between TC content and SSA are presented in Fig 3A. The mean values of TC and SSA obtained for soil variants with EOM were taken for analysis with the distinction of those values that come from the depths of 0–25 and 25–40 cm. Pearson’s correlation coefficients obtained for the particular soil depths were also estimated. TC showed a negative relation with SSA, R = -0.42 and R = -0.88, respectively, for the depth of 0–25 cm and 25–40 cm. Both coefficients were statistically significant at the level of significance α = 0.05. The dependence was much stronger for the soil depth of 25–40 cm and clearly showed preference and potential for the accumulation of carbon compounds in the deeper soil layer.

### Mercury intrusion porosimetry analysis

The porosity characteristics, V_MIP_, R_MIP_ and S_MIP_, are presented in Table 5. The parameters shown depend on the sampling depth as well as the type and dose of EOM used for soil fertilization. Samples taken from the depth of 0–25 cm exhibited a higher value of R_MIP_ and smaller values of S_MIP_, compared to samples taken from the depth of 25–40 cm. The values of R_MIP_, regardless of the soil depth, ranged from 80.88 nm to 417.25 nm, whereas S_MIP_ ranged from 2.06 m^2^/g to 9.66 m^2^/g. Cumulative pore volumes ranged from 0.37 to 0.44 cm^3^/g in the equivalent pore radius of 0.0015 to 180 μm.

**Table 5:** Pore parameters obtained for the studied variants in particular experiment periods.

	V_MIP_ (cm^3^·g^-1^)		S_MIP_ (m^2^·g^-1^)		R_MIP_ (nm)	
**Term**	**Control plot: 0–25 cm**					
**t**_**1**_	0.40±0.01		4.32±0.57		186.80±20.19	
**t**_**2**_	0.39±0.02		5.05±0.36		156.53±19.13	
**t**_**3**_	0.39±0.01		4.36±0.21		178.47±4.01	
**t**_**4**_	0.39±0.02		4.51±0.63		173.16±15.48	
**Term**	**Control plot: 25–40 cm**					
**t**_**1**_	0.38±0.02		8.93±0.45^ab^		85.83±8.81	
**t**_**2**_	0.38±0.03		8.64±0.25^b^		87.80±9.49	
**t**_**3**_	0.38±0.01		8.25±0.42^b^		91.94±7.11	
**t**_**4**_	0.39±0.01		9.66±0.27^a^		80.88±4.33	
	**PS: 0–25 cm**					
**Term**	**4.5 Mg/ha**	**9.0 Mg/ha**	**4.5 Mg/ha**	**9.0 Mg/ha**	**4.5 Mg/ha**	**9.0 Mg/ha**
**t**_**1**_	0.44±0.01	0.42±0.01^a^	2.19±0.17^b^	2.78±0.18	400.31±22.0^a^	304.15±12.42^a^
**t**_**2**_	0.41±0.02	0.37±0.01^b^	2.80±0.26^a^	2.74±0.23	297.22±13.37^c^	270.32±15.74^ab^
**t**_**3**_	0.41±0.01	0.39±0.00^b^	2.42±0.19^ab^	2.85±0.15	338.62±17.68^bc^	274.61±14.49^ab^
**t**_**4**_	0.42±0.01	0.42±0.01^a^	2.25±0.16^b^	3.14±0.24	378.80±18.13^ab^	266.42±14.15^b^
	**PS: 25–40 cm**					
**Term**	**4.5 Mg/ha**	**9.0 Mg/ha**	**4.5 Mg/ha**	**9.0 Mg/ha**	**4.5 Mg/ha**	**9.0 Mg/ha**
**t**_**1**_	0.41±0.01^ab^	0.38±0.01^b^	6.20±0.27^a^	7.08±0.68^a^	133.41±9.04^b^	107.74±13.29^b^
**t**_**2**_	0.41±0.01^b^	0.38±0.01^b^	3.29±0.21^c^	2.85±0.29^b^	248.21±22.00^a^	268.68±20.04^a^
**t**_**3**_	0.42±0.01^ab^	0.40±0.00^ab^	5.16±0.32^b^	6.31±0.23^a^	162.27±13.29^b^	125.79±4.59^b^
**t**_**4**_	0.44±0.02^a^	0.41±0.01^a^	6.70±0.19^a^	6.74±0.75^a^	131.98±2.23^b^	121.48±16.58^b^
	**DS: 0–25 cm**					
**Term**	**4.5 Mg/ha**	**9.0 Mg/ha**	**4.5 Mg/ha**	**9.0 Mg/ha**	**4.5 Mg/ha**	**9.0 Mg/ha**
**t**_**1**_	0.43±0.01^a^	0.43±0.01^a^	2.46±0.20^b^	2.06±0.15^c^	347.73±20.17^a^	417.25±21.14^a^
**t**_**2**_	0.40±0.02^b^	0.39±0.01^b^	3.03±0.22^a^	3.21±0.16^a^	267.38±24.74^b^	244.10±18.75^c^
**t**_**3**_	0.42±0.01^ab^	0.40±0.00^b^	2.55±0.18^ab^	2.18±0.15^bc^	328.79±15.82^a^	366.45±25.22^ab^
**t**_**4**_	0.43±0.01^a^	0.43±0.01^a^	2.63±0.24^ab^	2.55±0.21^b^	329.31±29.92^a^	337.96±20.57^b^
	**DS: 25–40 cm**					
**Term**	**4.5 Mg/ha**	**9.0 Mg/ha**	**4.5 Mg/ha**	**9.0 Mg/ha**	**4.5 Mg/ha**	**9.0 Mg/ha**
**t**_**1**_	0.41±0.01^ab^	0.40±0.01^b^	8.13±0.25^a^	8.99±0.54^a^	101.41±5.61^c^	88.74±7.57^c^
**t**_**2**_	0.38±0.01^b^	0.39±0.01^b^	4.34±0.21^d^	3.37±0.35^d^	173.47±12.95^a^	229.98±18.05^a^
**t**_**3**_	0.41±0.01^ab^	0.39±0.00^b^	5.55±0.33^c^	5.95±0.24^c^	146.98±12.30^ab^	130.95±5.29^b^
**t**_**4**_	0.44±0.02^a^	0.43±0.00^a^	7.06±0.16^b^	7.51±0.33^b^	125.99±8.56^bc^	114.06±5.02^bc^
	**FS: 0–25 cm**					
**Term**	**4.5 Mg/ha**	**9.0 Mg/ha**	**4.5 Mg/ha**	**9.0 Mg/ha**	**4.5 Mg/ha**	**9.0 Mg/ha**
**t**_**1**_	0.41±0.01	0.42±0.02^ab^	3.13±0.23^ab^	2.54±0.26^c^	264.00±12.99^a^	331.68±18.25^a^
**t**_**2**_	0.40±0.01	0.38±0.01^c^	3.72±0.28^a^	4.07±0.34^a^	218.59±21.79^b^	188.86±10.75^c^
**t**_**3**_	0.41±0.01	0.39±0.00^bc^	3.17±0.25^ab^	3.23±0.18^bc^	261.76±14.40^a^	242.83±13.56^b^
**t**_**4**_	0.42±0.00	0.43±0.01^a^	3.08±0.14^b^	3.58±0.30^ab^	274.87±12.36^a^	242.06±14.74^b^
	**FS: 25–40 cm**					
**Term**	**4.5 Mg/ha**	**9.0 Mg/ha**	**4.5 Mg/ha**	**9.0 Mg/ha**	**4.5 Mg/ha**	**9.0 Mg/ha**
**t**_**1**_	0.39±0.00^b^	0.40±0.01^ab^	5.96±0.17^bc^	8.60±0.25^a^	130.54±5.07	94.06±5.06^b^
**t**_**2**_	0.37±0.01^b^	0.39±0.01^ab^	5.75±0.22^c^	6.71±0.41^b^	130.37±9.17	118.02±10.21^a^
**t**_**3**_	0.40±0.01^b^	0.39±0.00^b^	6.26±0.23^b^	7.24±0.31^b^	128.61±8.25	108.10±4.63^ab^
**t**_**4**_	0.44±0.02^a^	0.41±0.01^a^	7.10±0.15^a^	7.48±0.36^b^	125.42±8.29	111.23±8.08^ab^

The table includes variants with the doses of 4.5 Mg/ha and 9.0 Mg/ha. No letter or the same letter indicates no significant differences between the values at the significance level α = 0.05, one-way ANOVA variance analysis, Tukey's HSD test. Abbreviations: PS—digestate from an agricultural biogas plant, DS—sludge from a dairy wastewater treatment plant, FS—sludge from a fruit wastewater treatment plant, t_1_-t_4_ –sampling periods, V_MIP_—total pore volume, S_MIP_—total pore area, R_MIP_—average pore radius.

**Fig 4:**
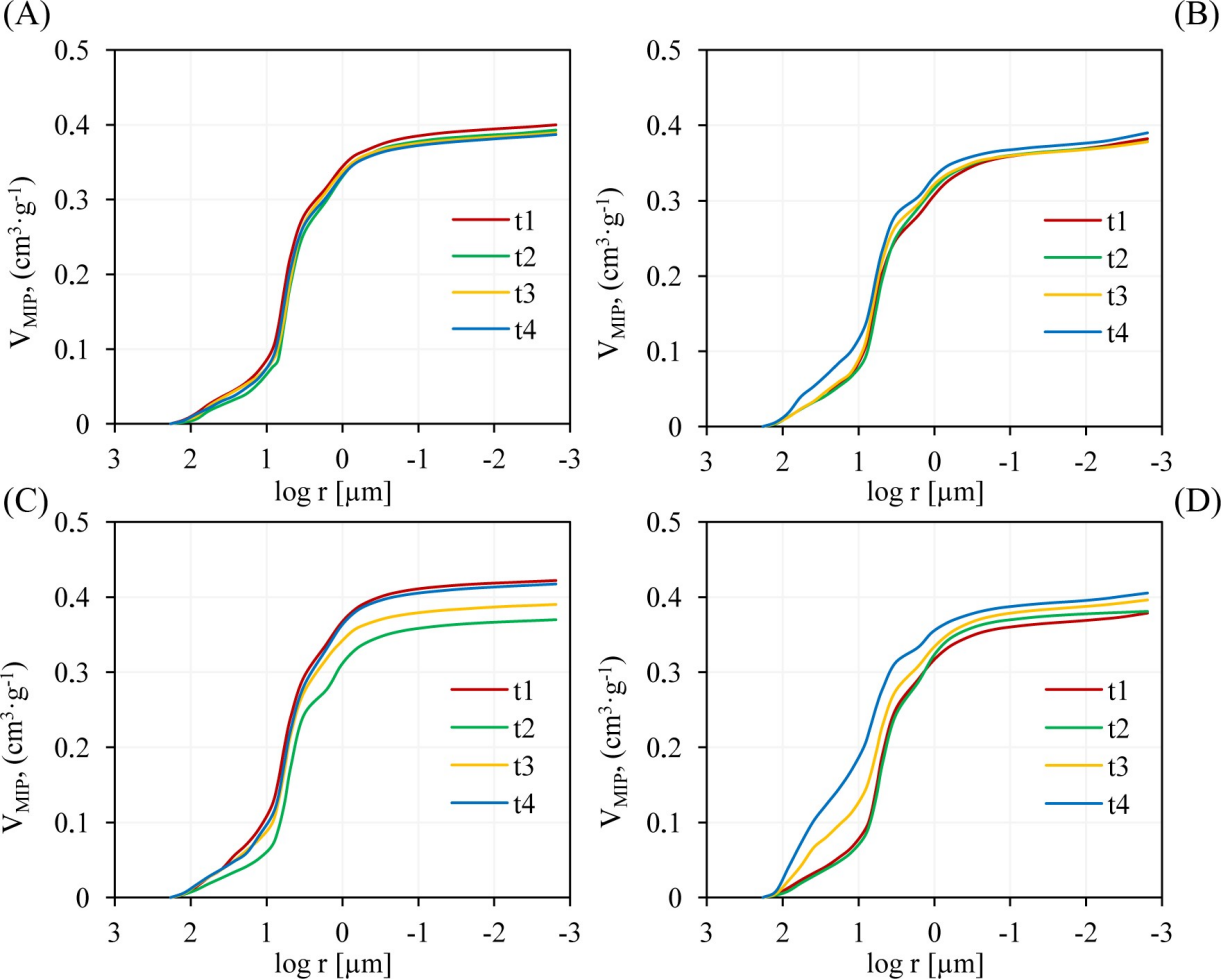
Mercury intrusion curves obtained for the control plot and variant with a higher dose of digestate from an agricultural biogas plant (PS). (A) control plot, soil depth 0–25 cm; (B) control plot, soil depth 25–40 cm; (C) PS, soil depth 0–25 cm, dose 9.0 Mg/ha; (D) PS, soil depth 25–40 cm, dose 9.0 Mg/ha. Abbreviations: t_1_-t_4_—means the period of sampling.

**Fig 5:**
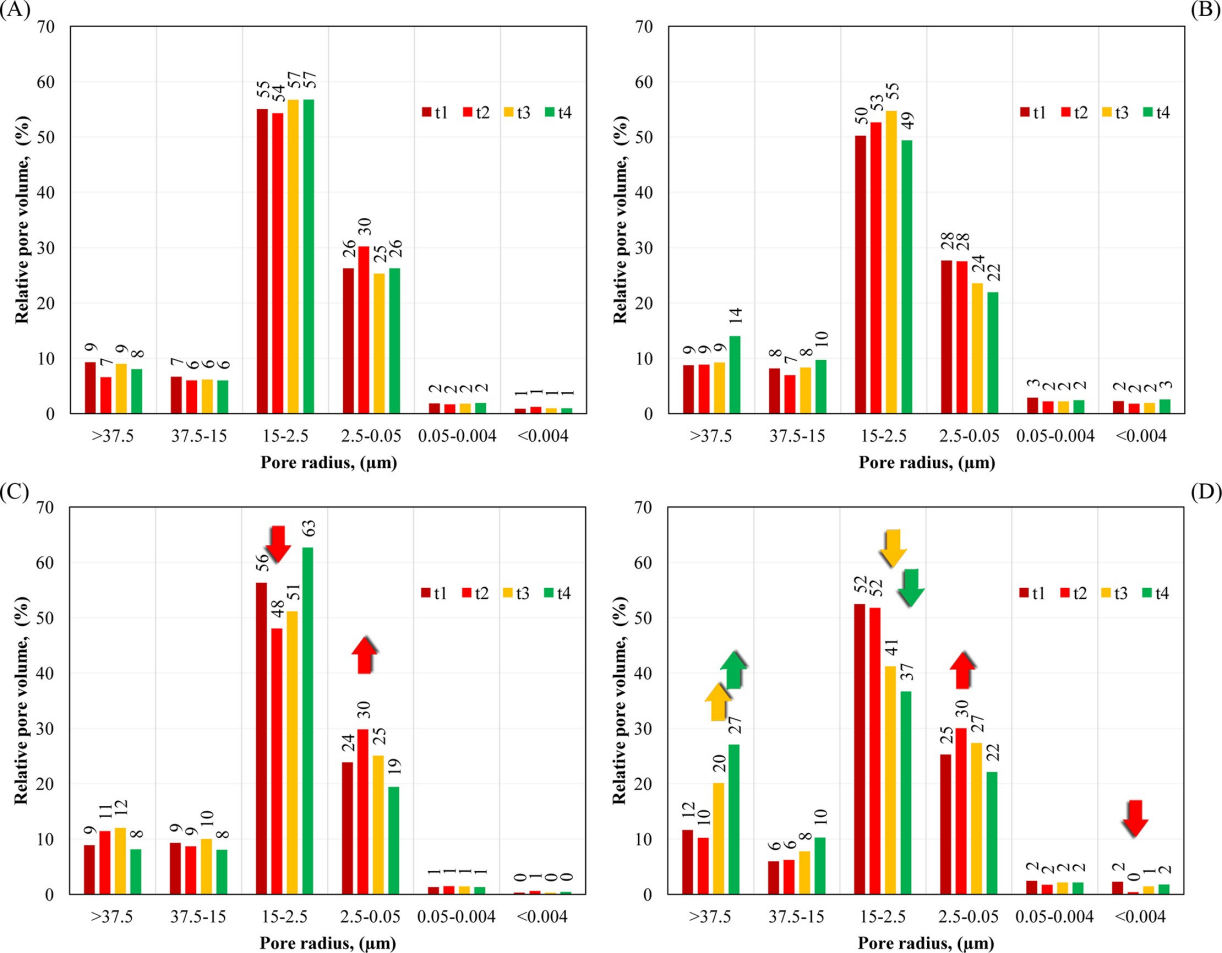
Relative porosity obtained for the control plot and variant with a higher dose of dairy sludge (DS). (A) control plot, soil depth 0–25 cm; (B) control plot, soil depth 25–40 cm; (C) DS, soil depth 0–25 cm, dose 9.0 Mg/ha; (D) DS, soil depth 25–40 cm, dose 9.0 Mg/ha. Abbreviations: t_1_-t_4_—means the period of sampling.

Dependence of the pore volume (cumulative) on the logarithm of their radius is shown in Fig 4 (see also S1 Fig). Sigmoidal curves start from the largest radius of pores on the X-axis so that the course of mercury intrusion can be followed upward from the lowest pressure. In the case of granular samples, at low pressure, mercury enters the pores between larger aggregates and into the large pores on aggregate surfaces. Then, as pressures increases, it penetrates the aggregate pore network as well as pores between smaller particles [37]. Relative pore size distributions are presented in Fig 5 (see also S2 Fig). To clarify the effect of EOM, the data was divided into five pore radius ranges (r > 37.5 μm; 37.5–15 μm; 15–2.5 μm, 2.5–0.05 μm, 0.05–0.004 μm and r < 0.004 μm) [38].

Almost all intrusion curves obtained for fertilized variants and a soil depth of 0–25 cm are below the control curve (t_1_) (Fig 4C, see also S1A and S1C Fig). The lower intrusion of mercury in the second experiment period (t_2_) was primarily associated with the lower number of pores in the range from 2.5 μm to 15 μm (DS variant) and those bigger than 15 μm (PS and FS variants) (Fig 5C, see also S2A and S2C Fig. The above effect was accompanied by the creation of pores of a smaller radius, namely in the range from about 0.05 μm to 2.5 μm. This resulted in R_MIP_ reduction from about 304 nm to 270 nm, 417 nm to 244 nm and from about 331 nm to 188 nm, respectively, for a higher dose of PS, DS and FS (Table 5). Except for a higher dose of PS, the differences were statistically significant at α = 0.05. S_MIP_ changed slightly indicating only minor impact of EOM on pores with the radius below 0.05 μm.

In t_3_ and t_4_, the reverse trend of intrusion was observed, suggesting the transient effect of EOM on the pore structure at the depth of 0–25 cm.

The effect of the application of EOM for the soil depth of 25–40 cm differs in comparison to that of 0–25 cm. With two exceptions (a higher dose of FS and a lower dose of DS), no significant changes were observed in the parameter of V_MIP_ in the second period of the experiment (t_2_) (Table 5). The S_MIP_ parameter decreased and R_MIP_ increased with TC. Calculated Pearson’s coefficients were statistically significant at the level of α = 0.05 and were R = -0.82 and R = 0.88 (p < 0.001), respectively for S_MIP_ and R_MIP_ (Fig 3C and 3D). Changes in S_MIP_ parameter were related to the lower number of pores of the < 0.004 μm radius. A higher dose of FS diminished the S_MIP_ parameter just to a minor extent, i.e. from 8.60 to 6.71 m^2^/g.

The most substantial changes in the shape of distribution of relative pore sizes were reported in the third (t_3_) and fourth period (t_4_) of the experiment for DS and PS variants. The reduction and shift of distribution towards the pores of a larger radius was observed (Fig 5D, see also S2B Fig). This was attributed to the lower number of pores in the radius range from about 0.05 μm to about 15 μm and was accompanied by the creation of an additional porosity in the range of pores of a radius greater than 15 μm.

## Discussion

### Exogenous organic matter accumulation dynamics

The application of organic wastes generally leads to an increase in the concentration of carbon compounds in soils which were indicated in numerous studies [11, 18, 19]. Some authors reported that organic wastes enhanced soil organic matter content proportionally to the application doses and/or frequency [11, 15, 17]. Field studies following a single application of organic waste also revealed an increase in soil organic matter content [5, 7].

Our results clearly showed that, irrespective of the EOM type and dose, the accumulation of carbon compounds at the soil depth of 0–25 cm was limited. This was unexpected but similar findings were obtained by other authors [20, 21, 39]. We suggest that this interesting effect could be related to mineral colloid surface saturation as well as to fast decomposition of organic material by microorganisms. The sorption of organic matter takes place at specific reactive sites of the mineral surface, such as edges, rough surfaces, or micropores [40]. When these reactive sites are occupied by native organic matter, exogenous compounds can have limited access to them. The binding strength of organic matter depends on the degree of coverage of mineral phases but also on the thickness of the sorption layer. In accordance with Kleber et al. [41], organic molecules become adsorbed in a layered structure on the mineral surfaces. The internal layers of organic matter are strongly bound to the mineral surface via ligand exchange and electrostatic attraction [42], thus providing less access by microorganisms [43]. In contrast, the external layers promote colonization by microorganisms that may cause partial degradation and mineralization of sorbed organic matter [44]. The mechanisms through which EOM can be biologically stabilized depend on the chemical structure of organic residues added to soil. EOM predominantly contains compounds of a small molecular weight and a low degree of humification. DS organic matter comes from the transformation of lactose, proteins, and fats [45], FS mainly from carbohydrates and cellulose [46], while in the case of PS mainly from lignins, and partially degraded proteins and cellulose [47]. This could partly explain why these numerous structures were susceptible to rapid decomposition and relocating to deeper soil layers.

EOM input has the potential to increase the pool of organic matter in the subsoil, with longer turnover time. Changes in the surface properties were related to coating and masking a large proportion of SSA. We found an inversely correlation between SSA and TC content. This effect can be supported by studies published previously [48, 49] that showed how the removal or addition of organic matter changes the external SSA of soil particles.

Our results indicate the presence of free highly developed surfaces in deeper soil layer. These structures, in the form of aluminosilicates, Fe and Al oxides, allow a physical protection of dissolved organic matter by encapsulation within soil aggregates or creation of organo-mineral associations. This stay in line with McCarthy et al. [50] that observed encapsulation of colloidal organic matter by minerals after long-term field manipulations of land use and agricultural management. In turn, Eusterhues et al. [51] showed the importance of Fe and Al oxides in the formation of organo-mineral associations in subsoils. Authors suggested that the resulting large amount of oxide-specific reactive surface sites was responsible for their dominant role as sorbents.

### Environmental effect of exogenous organic matter on pore size evolution

Soil pores are arranged hierarchically with the primary (textural) and secondary (structural) pore system [52]. Bigger pores attributed to biological activity, climate, and management practices are called structural pores and are also referred to as inter-aggregate pores. In contrast, textural porosity refers to pores within aggregates and between primary soil particles. These kinds of structures are also known as matrix, intra-aggregate, and inter-particle pores [28].

Our results clearly showed that the application of exogenous materials influenced soil porosity. We suppose that at a soil depth of 0–25 cm, high hydration of organic waste, organic particle mineralization and/or migration contributed to a major reorganization of the pore space.

EOM reduced mainly the volume of pores with a radius from 2.5 μm to 15 μm. In some cases, (for PS and FS), a decrease in the number of transmission pores was also observed. These pores correspond to inter-aggregates pores, as well as the arrangement of micro-aggregates between primary soil particles [38]. In terms of function, they provide water and nutrients for plants and microorganisms [53] and could be the main route of migration of some heavy metals [54]. Less number of storage and transmission pores means that some soil processes, such as drainage, aeration or plant growth could be impeded after EOM application. Kuncoro et al. [29] observed the diminish of air permeability and relative gas diffusivity for the soil with applied organic matter. Authors suggested that the presence of organic matter blocked soil pores and increased the capillary water in the pore necks. According to Schneider et al. [55], coarse organic matter fraction caused occlusion of pores and increased the hydrophobicity of soil components leading to a decrease in soil hydraulic conductivity. A similar effect on soil hydraulic conductivity was observed after the initial addition of green-waste based composts to soils with low and high organic matter content [56].

It is worth noting that the decreasing volume of larger pores was accompanied by the rising volume of fine pores in the radius range of 0.05 μm to 2.5 μm. This interrelation of the pore size ranges leads to a greater number of contact points between soil particles and may temporarily increase the mechanical strength of soil aggregates [57].

Despite the fact that EOM diminished the volume of pores at the soil depth of 0–25 cm, we did not find any considerable correlation between V_MIP_ and TC content, R = -0.32 (Fig 3B). We suppose that our finding is related to native carbon attached to soil mineral phase. EOM affected only pores which contribute to the pore volume heavily but also exhibit a little carbon storage potential. This could be also the reason of decrease in R_MIP_ and insignificant correlation of this parameter with TC, R = -0.21 (Fig 3D).

At the depth of 25–40 cm, the application of EOM initially decreased the volume of pores with a radius of <0.004 μm. This was probably related to the occlusion of micro- aggregates as well as fine silt-sized and clay-sized loose particles that contain phases of high microporosity [50]. In line with Kaiser et al. [40] micropores are lost in the initial stages of the mineral surface accumulation of organic matter. Similar results were obtained by Benito et al. [12] that investigated the effects of industrial sludge application on soil pores using N_2_ adsorption and time-domain magnetic resonance relaxometry. Authors reported that pores <2 nm in diameter disappeared and larger pores were clogged to some extent.

In the following periods of the experiment, for PS and DS variants, an increase in the number of structural pores (>15 μm in pore radius) was found. These results could be supported by De Gryze et al. [32] that revealed an increase in soil void porosity (27–67 μm diameter range) after application of fresh organic residue. Authors added that pore creation could result from different mechanisms involving: i) shrinking and swelling of soil and organic particles as a response to wetting and drying cycles ii) decomposition of organic matter by microorganisms, iii) fungal hyphae creation that pushing aside silt-sized particles. Similar findings were also obtained by Dlapa et al. [28], who reported that a higher carbon content promotes the formation of elongated pores with a characteristic pore diameter of 15–27 μm. Authors added that creation of micro-crack improves soil hydraulic properties due to the higher ability of structural pores to drain water during the rainfall.

In our studies, the creation of structural porosity was linked with a simultaneous decrease in TC content and could indicate an early stage of aggregate formation. In this process, organic matter binds fine mineral particles into clusters and then breaks down within the macroaggregates [58]. Aggregation increases with soil depth and produces large, irregularly distributed units which in later stages of development are separated by cracks [59]. The above processes were clearly described by Grosbellet et al. [8] that studied the effects of the evolution of a large quantity of organic matter on the aggregation dynamics and structural characteristics of the soil. Authors showed preferential carbon sequestration in the macroaggregates that led to the modification of the aggregate organisation with the presence of elongated pores. It is worth noting that in the case of FS, we did not observe formation of elongated pores, probably due to the lowest content of organic compounds. We suggest that in the case of FS application, the thickness of the layer covering the mineral surfaces was insufficient to create organic matter bonds between fine particles and to allow these particles to be taken-up within aggregates.

### Practical implications of the study

Our study showed that the direct use of sludge on agricultural land as an organic amendment is a good recycling option. As organic waste is an inexpensive source of organic matter, it can be used as a soil conditioner to restore eroded, degraded agricultural land that is poor in organic matter. In this context, the uses of organic waste can partially replace traditional mineral and organic fertilizers result in the improvement of physicochemical properties of soil at lower costs. Our result also implies that organic waste application could be of great importance in case of heavy clay soils and subsoils that tend to have poor drainage, and become compacted easily. The creation of new structural porosity may provide a high rate of rainfall infiltration to this soils and reduce their susceptibility to surface runoff and accompanying erosion.

We also postulate that long-lasting improvement of the soil physical status can be obtained by successive and moderate applications of organic sludge. As was demonstrated, the exogenous organic matter might not be stabilized in the long-term period if it is not protected by physical or chemical mechanisms against microbial degradation. We recommend the use of additional mineral sorbents together with organic wastes to support the accumulation of carbon compounds and help to regulate hydraulic properties in the topsoil.

Our research has also shown the practical usefulness of mercury porosimetry and nitrogen adsorption-desorption methods in the assessment of the relationships between soil organic carbon and porosity and the effect of the organic waste application. The approach used and results of this study can help to improve the multi-domain simulation of water, solute flow, and pore-size dependent sorption.

## Conclusions

Our results imply that soil fertilization with organic materials of different origin influenced the surface characteristics of the soil. The intensity of observed changes depended on the type and dose of organic waste, soil depth and sampling date. The decrease in SSA as a result of the accumulation of exogenous organic matter was observed only at a soil depth of 25–40 cm. We link this effect with a higher number of reactive sites and thus a higher potential of deeper soil layer for the organic matter preservation.

As demonstrated, pore size distributions of the soil changed as a result of the evolution of added organic matter. Organic wastes application affected most of the tested pore characteristics such as the total pore volume, average pore radius and total pore area. The most interesting observations showed an increase in the volume of elongated pores in the deeper soil layer. We concluded that this new structural porosity was associated with the breakdown of organic particles when forming stable aggregates.

Future research is necessary for developing sustainable management practices of organic waste in agricultural land. These studies could focus on the following issues: the mechanisms of soil structure formation—identification of root growth dynamics and soil fauna interactions; the use of co-fertilization by organic waste and mineral sorbents in the context of carbon preservation; the creation of new organo-mineral complexes in the subsoil.

## Supporting information

S1 Fig Mercury intrusion curves obtained for variants with a higher dose of dairy sludge (DS) and fruit sludge (FS). (A) DS, soil depth 0–25 cm, dose 9.0 Mg/ha; (B) DS, soil depth 25–40 cm, dose 9.0 Mg/ha; (C) FS, soil depth 0–25 cm, dose 9.0 Mg/ha; (D) FS, soil depth 25–40 cm, dose 9.0 Mg/ha. Abbreviations: t_1_-t_4_—means the period of sampling.
(TIF)

S2 Fig Relative porosity obtained for variants with a higher dose of digestate (PS) and fruit sludge (FS). (A) PS, soil depth 0–25 cm, dose 9.0 Mg/ha; (B) PS, soil depth 25–40 cm, dose 9.0 Mg/ha; (C) FS, soil depth 0–25 cm, dose 9.0 Mg/ha; (D) FS, soil depth 25–40 cm, dose 9.0 Mg/ha. Abbreviations: t_1_-t_4_—means the period of sampling.
(TIF)
