# Supplementary figures and images for: The effect of application of digestate and agro-food industry sludges on Dystric Cambisol porosity
Source: PLoS One. 2020 Sep 2;15(9):e0238469. doi: 10.1371/journal.pone.0238469 (PMC7467315; doi:10.1371/journal.pone.0238469)

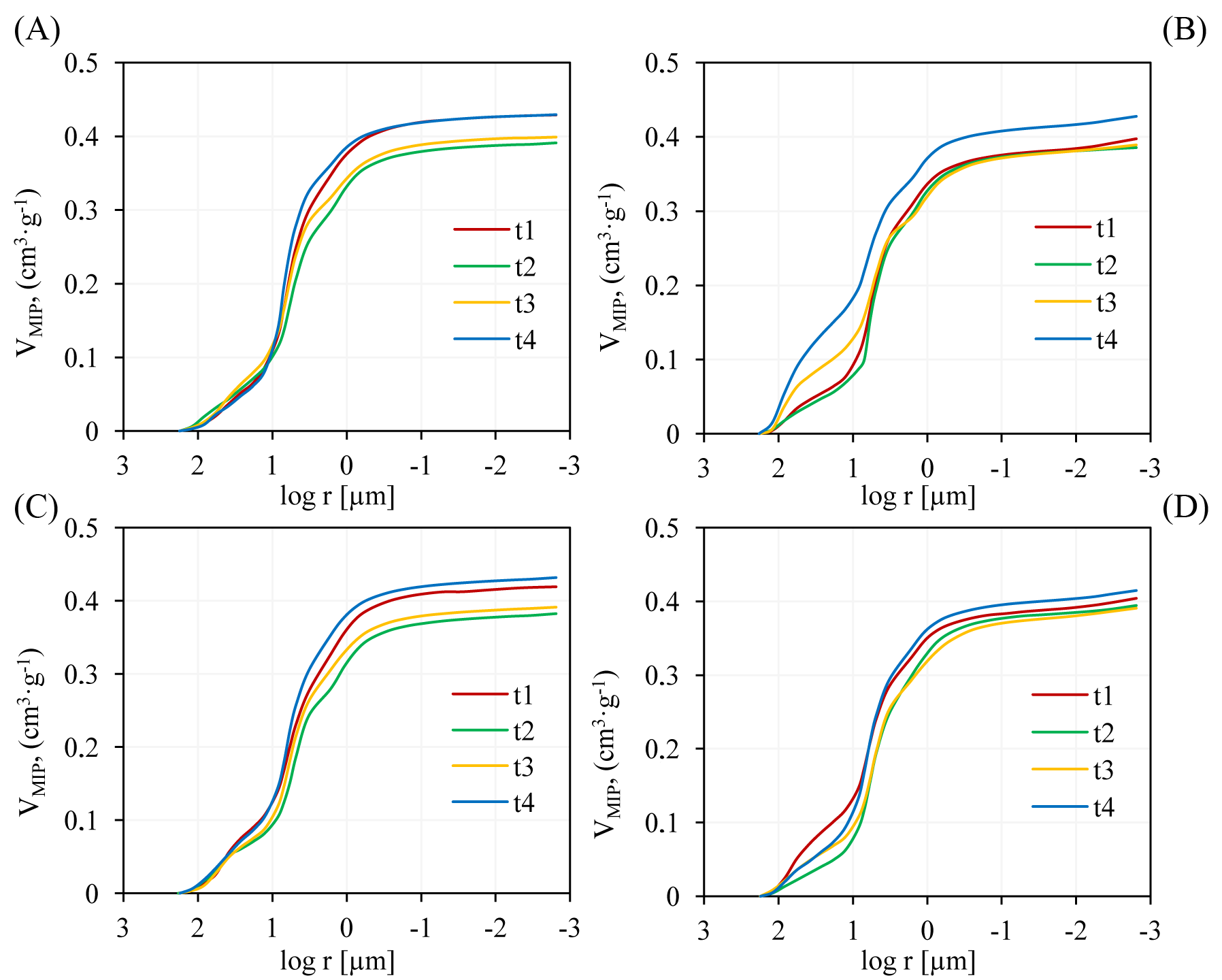

Supplement: S1 Fig — (A) DS, soil depth 0–25 cm, dose 9.0 Mg/ha; (B) DS, soil depth 25–40 cm, dose 9.0 Mg/ha; (C) FS, soil depth 0–25 cm, dose 9.0 Mg/ha; (D) FS, soil depth 25–40 cm, dose 9.0 Mg/ha. Abbreviations: t1-t4—means the period of sampling. (TIF) [file pone.0238469.s001.tif]

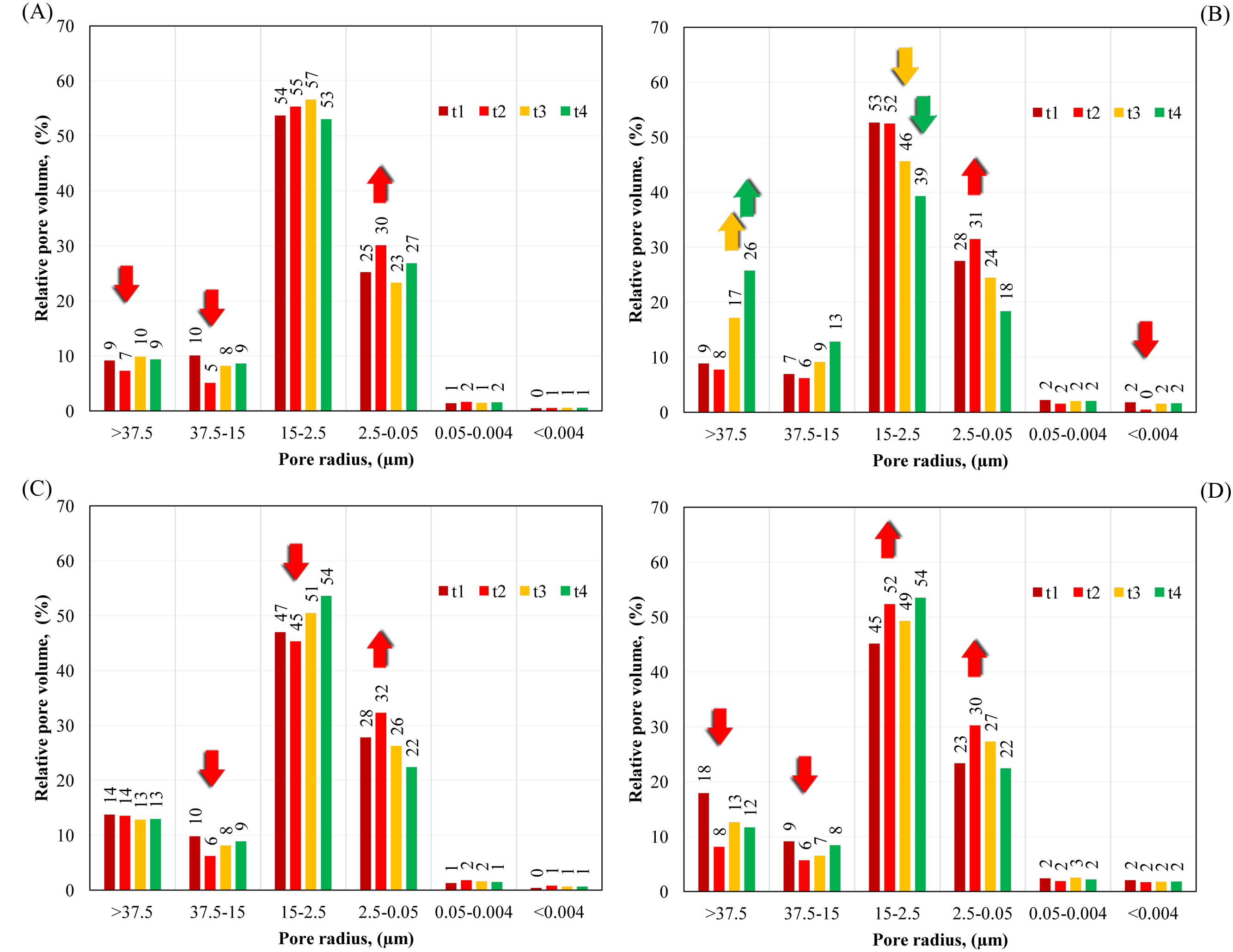

Supplement: S2 Fig — (A) PS, soil depth 0–25 cm, dose 9.0 Mg/ha; (B) PS, soil depth 25–40 cm, dose 9.0 Mg/ha; (C) FS, soil depth 0–25 cm, dose 9.0 Mg/ha; (D) FS, soil depth 25–40 cm, dose 9.0 Mg/ha. Abbreviations: t1-t4—means the period of sampling. (TIF) [file pone.0238469.s002.tif]
